# Regulation of Neurite Growth by Inorganic Pyrophosphatase 1 via JNK Dephosphorylation

**DOI:** 10.1371/journal.pone.0061649

**Published:** 2013-04-23

**Authors:** Yu Tezuka, Mizuki Okada, Yuka Tada, Junji Yamauchi, Hideo Nishigori, Atsushi Sanbe

**Affiliations:** 1 Department of Pharmacotherapeutics, School of Pharmacy, Iwate Medical University, Iwate, Japan; 2 Department of Pharmacology, National Research Institute for Child Health and Development, Tokyo, Japan; Institut National de la Santé et de la Recherche Médicale (INSERM U901), France

## Abstract

Neural cell differentiation during development is controlled by multiple signaling pathways, in which protein phosphorylation and dephosphorylation play an important role. In this study, we examined the role of pyrophosphatase1 (PPA1) in neuronal differentiation using the loss and gain of function analysis. Neuronal differentiation induced by external factors was studied using a mouse neuroblastoma cell line (N1E115). The neuronal like differentiation in N1E115 cells was determined by morphological analysis based on neurite growth length. In order to analyze the loss of the PPA1 function in N1E115, si-RNA specifically targeting PPA1 was generated. To study the effect of PPA1 overexpression, an adenoviral gene vector containing the PPA1 gene was utilized to infect N1E115 cells. To address the need for pyrophosphatase activity in PPA1, D117A PPA1, which has inactive pyrophosphatase, was overexpressed in N1E115 cells. We used valproic acid (VPA) as a neuronal differentiator to examine the effect of PPA1 in actively differentiated N1E115 cells. Si-PPA1 treatment reduced the PPA1 protein level and led to enhanced neurite growth in N1E115 cells. In contrast, PPA1 overexpression suppressed neurite growth in N1E115 cells treated with VPA, whereas this effect was abolished in D117A PPA1. PPA1 knockdown enhanced the JNK phosphorylation level, and PPA1 overexpression suppressed it in N1E115 cells. It seems that recombinant PPA1 can dephosphorylate JNK while no alteration of JNK phosphorylation level was seen after treatment with recombinant PPA1 D117A. Enhanced neurite growth by PPA1 knockdown was also observed in rat cortical neurons. Thus, PPA1 may play a role in neuronal differentiation via JNK dephosphorylation.

## Introduction

Neural cell differentiation during development includes outgrowth of neurites, which later become axons and dendrites, and is controlled by multiple signaling pathways in which protein phosphorylation and dephosphorylation play an important role [1]–[3]. Polarized neurons have a single axon and some dendrites, and can form synaptic contacts to establish their networks [4], [5]. During neurite growth, dynamic remodeling of the cytoskeleton is required for these morphological and biochemical changes to occur [4]. However, the initial steps of the neurite growth mechanism are not fully understood, and there is growing evidence regarding the signaling pathways responsible for neuronal polarity and synaptic formation [4]–[6].

Modification of actin cytoskeleton proteins by signaling cascades such as mitogen-activated protein kinases (MAPKs), are the direct regulators of the actin cytoskeleton [7], [8]. Many previous studies indicate that the process of neurite extension is generally regulated by Rac1 and Cdc42 activities, subsequent activation of c-Jun N-terminal kinase (JNK; a subfamily of MAPK), and phosphorylation of paxillin [9]–[12]. We showed that paxillin phosphorylation, acting through the Rac1/Cdc42/cJNK signaling cascade, is activated following neurite extension in mouse N1E115 neuroblastoma cells [11]. In addition, we also reported that valproic acid (VPA), a short-branched fatty acid used as a mood-stabilizing agent for the treatment of manic-depressive illness (also known as bipolar disorder) and as an anticonvulsant [13], [14], can promote neurite outgrowth via the JNK activation in mouse neuroblastoma N1E115 cells [10], [11]. Thus, the JNK phosphorylation of paxillin, possibly after Rac1/Cdc42 signaling cascade stimulation, plays a critical role in neurite extension in mouse N1E115 neuroblastoma cells [11]. Although numerous studies have explored phosphorylation of JNK, the regulation of neuronal differentiation, particularly related to protein dephosphorylation via protein phosphatase, remains uncertain.

Inorganic pyrophosphates are generated as byproducts of many biosynthetic reactions, including DNA and RNA synthesis, fatty acid and amino acid activation, and cyclic nucleotide synthesis [15]–[18]. Inorganic pyrophosphatase 1 (PPA1) is thought to play a role in catalyzing the hydrolysis of pyrophosphates into organic phosphates, which are then exported across the cell membrane [15]. However, physiological role of PPA1 in neuronal tissue, particular during neuronal development, is uncertain.

In this study, we examined the role of PPA1 in neuronal differentiation by the loss and gain of function analysis using N1E115 cells. Our results suggest that PPA1 may play a role in neuronal differentiation, such as neurite growth, as a protein phosphatase via JNK dephosphorylation.

## Methods

### cDNAs

PPA1 cDNA was isolated using reverse transcription-PCR and used to generate recombinant protein and adenoviral constructs as described previously [19], [20]. Since aspartic acid at position 117 in PPA1 is important for its enzymatic activity, the missense mutation PPA1 Asp117Ala (D117A), which result in an inactive form of pyrophosphatase activity [21], [22], were introduced using reverse transcription-PCR and subcloned into the pBSKII vector (Agilent Technologies, Palo Alto, CA, USA).

### Recombinant protein

To produce the recombinant protein, His epitope-tagged PPA1 and PPA1 D117A were overexpressed in BL21 cells (Invitrogen, Carlsbad, CA, USA) using the pET system (Novagen, Madison, WI, USA) and purified using a Ni-NTA column (Qiagen, Santa Clarita, CA, USA) as described previously [19].

### Pyrophosphatase activity

Recombinant protein pyrophosphatase activity was determined using Molybdate Dye solution (Promega, Fitchburg, WI) [23], [24]. A mixture containing 50 mM pyrophosphatase and 20 µg recombinant PPA1 or PPA1 D117A protein was incubated at 37°C for 30 min, and the presence of inorganic phosphate released was determined using Molybdate Dye solution.

### siRNA oligonucleotides

The 21-nucleotide siRNA duplexes were synthesized using Nippon Gene Material Co.,Ltd. (Toyama, Japan), and we designed the target nucleotide sequences, 5′-AAGGATGTGTTCCACATGGTG -3′ for mouse and rat PPA1 siRNA. The target sequence of the control Photinus pyralis luciferase siRNA was 5′-AAGCCATTCTATCCTCTAGAG-3′, which has no significant homology to any mammalian gene sequence.

### siRNA transfection

Cells were transfected with siRNA oligonucleotides using the Lipofectamine 2000 reagent (Invitrogen), according to the manufacturer's protocol.

### RNA preparation and RT-PCR analysis

Total RNA from N1E115 cells was prepared using ISOGEN reagent (NIPPON GENE, Toyama, Japan). The cDNAs were prepared from 1 µg of total RNA using PrimeScript reverse transcriptase (Takara Bio, Kyoto, Japan), according to the manufacturer's instructions. PCR amplification was carried out using ExTaq polymerase (Takara Bio) at 30 cycles, each cycle consisting of denaturation at 94°C for 0.5 min, annealing at 60°C for 0.5 min, and extension at 72°C for 1 min. Quantitative realtime-PCR (RT-PCR) was performed using the Eco™ RT-PCR system (illumine, San Diego, CA, USA) according to the manufacturer's protocol. The primers used were 5′- TGCTGCCVAAAGCCATTGTGGATG-3′ (sense) and 5′-TCAGTTTTTCTGCTGATGGAAC -3′ (antisense) for mouse PPA1; 5′-AGGTCATCCATGACAACTTTG-3′ (sense); and 5′- TTCAGCTCTGGGATGACCTT-3′ (antisense) for mouse GAPDH.

### N1E115 cell culture and adenovirus infection

Mouse neuroblastoma N1E115 cells that were originally purchased from DS Pharma Biomedical Co. Ltd (Osaka, Japan) were kindly given by Dr. S. Tanuma (Department of Pharmaceutical Sciences, Tokyo University of Science) and were cultured as described previously [11]. The cells were infected at an infection multiplicity of 100 for each virus. Replication-deficient recombinant adenoviruses containing the mouse PPA1 gene were made using an AdEasy system (Agilent Technologies), as described previously [19], [25], [26]. The adenovirus-containing green fluorescent protein (GFP) gene was used as a control [27]. In our pilot study using adenoviral vector-containing GFP, almost 100% of N1E115 cells were infected and expressed the GFP gene 12 hr after infection.

In our previous study, we showed that VPA can promote neurite outgrowth via the JNK activation in mouse neuroblastoma N1E115 cells [10], [11]. Thus, in order to examine the effect of PPA1 in actively differentiated N1E115 cells, we used VPA as a neuronal differentiator in this study [10], [11].

### Rat cortical neurons

Cerebral cortical neurons were isolated from E18.5 Sprague-Dawley (SD) rat fetuses [28]. The brains were removed, and the neocortices were dissected out. The neocortices were enzymatically dissociated with 0.05% trypsin (Life Technologies Corporation, Carlsbad, CA), and 5×10^5^ cells/cm^2^ were grown in serum-free DMEM on collagen-coated dishes at 37°C containing 5% CO_2_. After 180 min, the plating medium was aspirated and replaced with a serum-free defined medium consisting of Neurobasal, with 2% B27 supplement, 0.1 mg/ml Gentamicin (Life Technologies Corporation, Carlsbad, CA). Cells were randomly selected, and the number of neurites was counted using microscopy. To determine the involvement of JNK activity in neurite growth in the rat neuron, the effect of 10 µM SP600125, a JNK inhibitor (Calbiochem-Novabiochem CA), was used [11].

### Miscellaneous methods

Cellular proliferation was measured following bromodeoxyuridine (BrdU) incorporation [27]. Cultures were incubated with 10 mM BrdU for 2 hr. Cells were then fixed and stained for BrdU using streptavidin–biotin (Invitrogen), and labeled cells were counted using Image J1.38× public domain software. Sample preparation for Western blotting, gel preparation, and electrophoretic conditions were carried out as described previously [26], [27]. Western blot analyses were performed using anti-PPA1 antibody (ab58134, Abcam, Cambridge, UK), anti-Akt antibody (9272, Cell Signaling Technology, Inc., Danvers, MA), anti-(pSer473)AKT antibody (anti-phospho-Akt antibody) (9217, Cell Signaling Technology, Inc), anti-p38 MAPK antibody (9212, Cell Signaling Technology, Inc.), anti-(pThr180/Typ182)p38 MAPK antibody (anti-phospho-p38 MAPK antibody) (4631, Cell Signaling Technology, Inc.), anti-Erk1/2 antibody (4695, Cell Signaling Technology, Inc.), anti-(pThr202/Typ204)Erk antibody (anti-phospo-Erk1/2 antibody) (4370, Cell Signaling Technology, Inc.), anti-SAPK/JNK antibody (9258, Cell Signaling Technology, Inc.), anti-(pThr183/Typ182)-SAPK/JNK antibody (anti-phospho-SAPK/JNK antibody) (4668, Cell Signaling Technology, Inc.), anti-GAPDH antibody (Chemicon International, Temecula, CA, USA), anti-Paxillin antibody (610051, BD Transduction Laboratories, Franklin Lakes, NJ), and anti-(pSer178)paxillin antibody (anti-phospho-paxillin antibody) (44-1026G, Life Technologies Corporation, Carlsbad, CA). The band intensity in the immunoblot was semi-quantified using Image J1.38x public domain software. Immunohistochemical analyses were performed as described previously [27]. Samples were incubated with the anti-PPA1 antibody (ab58134, Abcam, Cambridge, UK) at 4°C for 1.5 hr. All pictures were taken under the same experimental conditions including exposure time.

### Immunoprecipitation assay

Immunoprecipitation was performed as described previously [19]. Cells were scraped into 0.5 ml TBS buffer [19], and the extracts were vortexed and centrifuged at 18,000 *g* for 15 min at 4°C. The supernatants were mixed with 1.5 volumes of a TBS buffer and an anti-JNK antibody (9258, Cell Signaling Technology, Inc.), and protein A/G plus agarose immunoprecipitation reagent (sc-2003, Santa Cruz Biotechnology, Inc., Santa Cruz, CA) followed by incubation at 4°C overnight. The reagent was washed three times using TBS buffer. To determine the effects of recombinant protein such as his-PPA1 and his-PPA1 D117A on phosphorylated-JNK or phosphorylated-paxillin, 10 µl of immunoprecipitated sample obtained using anti-JNK antibody was incubated with 20 µg recombinant PPA1 or PPA1 D117A protein at 37°C for 2 hr.

The complexes were analyzed using Western blot after SDS-PAGE as described above [26], [27].

### Statistics

Data are expressed as the mean ± standard error. Statistical analysis was performed using the unpaired Student's T-test and one-way ANOVA followed by a post hoc comparison using Scheffe's multiple comparison. Statistical tests were performed using Kaleida Graph version 4.1 software (Synergy Software, Reading, PA, USA).

### Ethics

This study was approved by the Animal Care Committee of Iwate Medical University. All experimental procedures were performed in accordance with the Guidelines of the Iwate Medical University Ethics Committee for Animal Treatment and the Guidelines for Proper Conduct of Animal Experiments by the Science Council of Japan.

## Results

### PPA1 loss- and gain-of-function analysis in N1E115 cells

**Figure 1:**
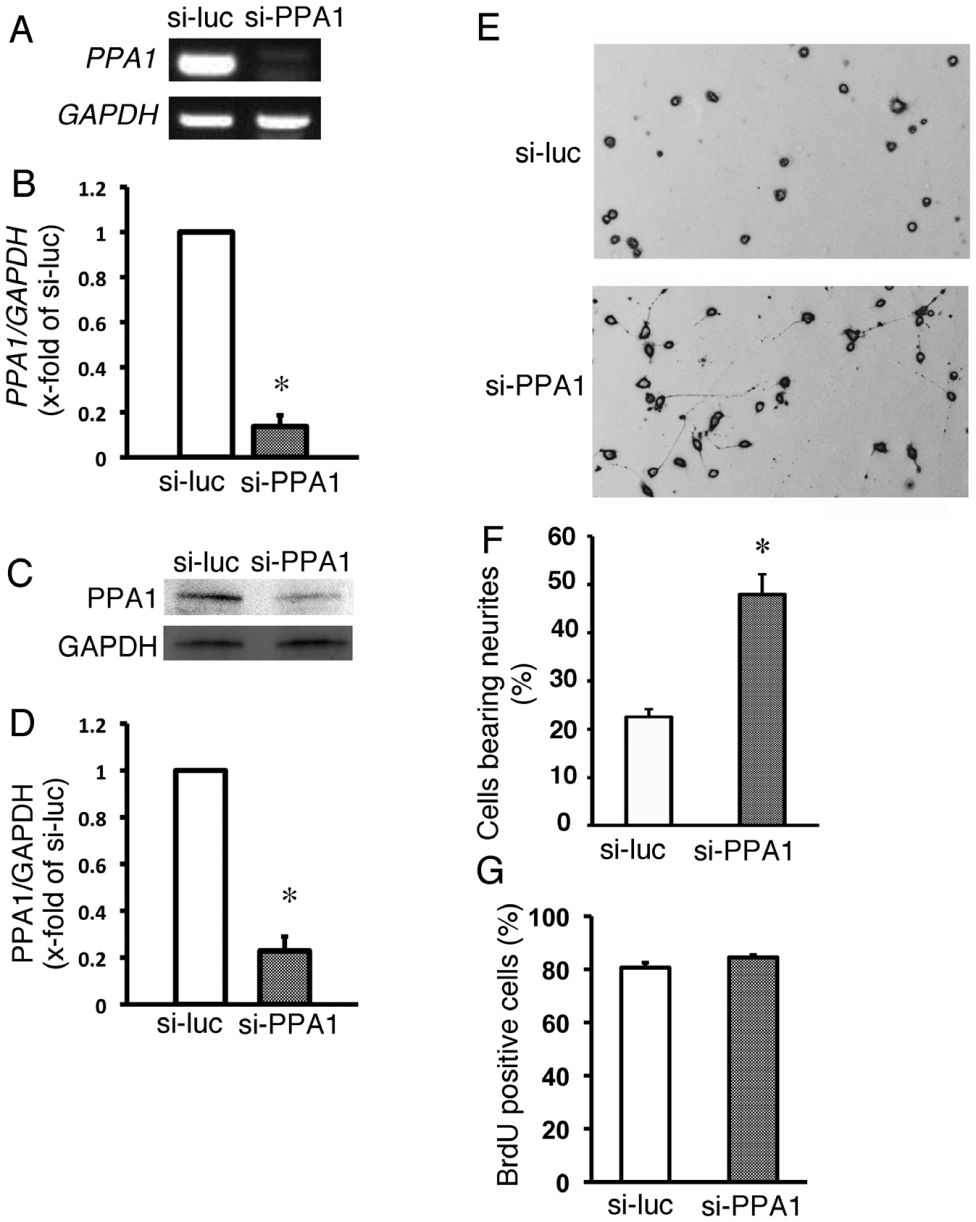
Loss-of-function PPA1 analysis in N1E115 cells. (A and B) Mouse PPA1 knock-down in N1E115 cells using siRNA targeted to mouse PPA1 (si-PPA1). si-RNA targeted to luciferase (si-Luc) was used as a control. Representative RT-PCR (A) and quantitative analysis of PPA1 expression using real time PCR (B). Representative Western blot analysis (C) and quantitative analysis of PPA1 protein (D). Values are the fold increase relative to the si-Luc, with its value arbitrarily set to 1 (n = 5). (E) Representative N1E115 cells treated with si-RNA targeted to mouse PPA1 and targeted to luciferase as a control (E). (F) Quantitative analysis of the neurite growth ratio is shown. Neurite growth is determined by morphological analysis as described in the Experimental procedure. (n = 100) (G) N1E115 cell proliferation. Cell proliferation was measured using bromodeoxyuridine (BrdU) incorporation into the cells (n = 5). *p<0.05 vs. si-Luc.

In this study, we examined the functional role of PPA1 during neuronal like differentiation using N1E115 cells [11], [29]. PPA1 gene expression and its protein level in N1E115 cells treated with siRNA targeted to PPA1 was measured using realtime-PCR and Western blot (Figure 1A–D). PPA1 expression (Figure 1A–B) and protein (Figure 1C–D) levels were dramatically reduced by treatment with the PPA1-specific siRNA compared to that in N1E115 cells treated with the luciferase-specific siRNA. Neurite extension was enhanced in the N1E115 cells (Figure 1E–F). PPA1 knock-down did not include any alteration in cellular proliferation, as determined by BrdU incorporation in N1E115 cell (Figure 1G). Thus, these results suggest that PPA1 knock-down enhances neurite growth in the N1E115 cells.

**Figure 2:**
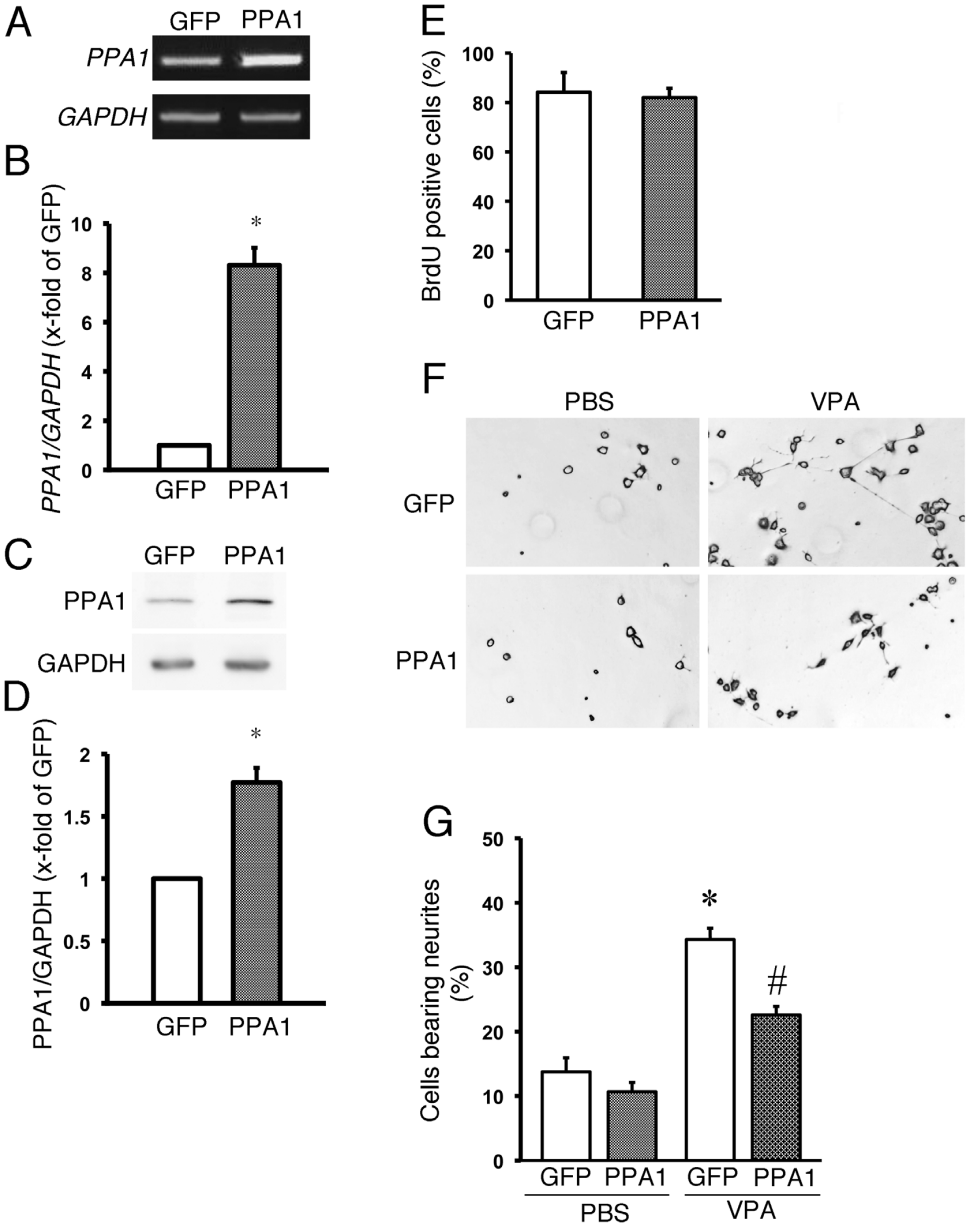
Gain-of-function PPA1 analysis in N1E115 cells. (A and B) Mouse PPA1 overexpression in N1E115 cells using adenoviral vector (AD-PPA1). An adenoviral vector containing green fluorescence protein (GFP) was used (AD-GFP) as a control. Representative RT-PCR (A) and quantitative PPA1 expression analysis using real time PCR (B). Representative Western blot analysis (C) and quantitative analysis of PPA1 protein (D). Values are the fold increase relative to the AD-GFP, with its value arbitrarily set to 1 (n = 5). (E) N1E115 cell proliferation was measured using bromodeoxyuridine (BrdU) incorporation into the cells (n = 5). (F) Typical morphological changes of in N1E115 cells treated with 1 mM valproric acid (VPA), a stimulator of neuronal differentiation in N1E115 cells, by using AD-GFP and AD-PPA1. Overexpression of AD-GFP was used as a control. (G) Quantitative analysis of the neurite growth ratio is shown. Neurite growth is determined by morphological analysis as described in the Experimental procedure (n = 100). *p<0.05 vs. AD-GFP.

We then analyzed the effect of PPA1 overexpression in N1E115 cells. An increase in PPA1 gene expression and protein levels were detected in the N1E115 cells treated with adenovirus containing the mouse PPA1 gene (Figure 2A–D). No difference in cellular proliferation was observed in the N1E115 cells between PPA1-overexpressing and GFP-overexpressing N1E115 cells (Figure 2E), whereas PPA1 overexpression showed inhibitory effects of the neurite growth in the N1E115 cells stimulated by VPA (Figure 2F and G). Since PPA1 overexpression can inhibit the neurite growth in activated N1E115 cells treated with VPA, and PPA1 knock-down can enhance the neurite growth in N1E115 cells, these results indicate that PPA1 can play a critical role in neurite growth and may work as an inhibitor of neuronal differentiation in N1E115 cells.

### Pyrophosphatase activity is necessary for neurite growth inhibition in N1E115 cells

**Figure 3:**
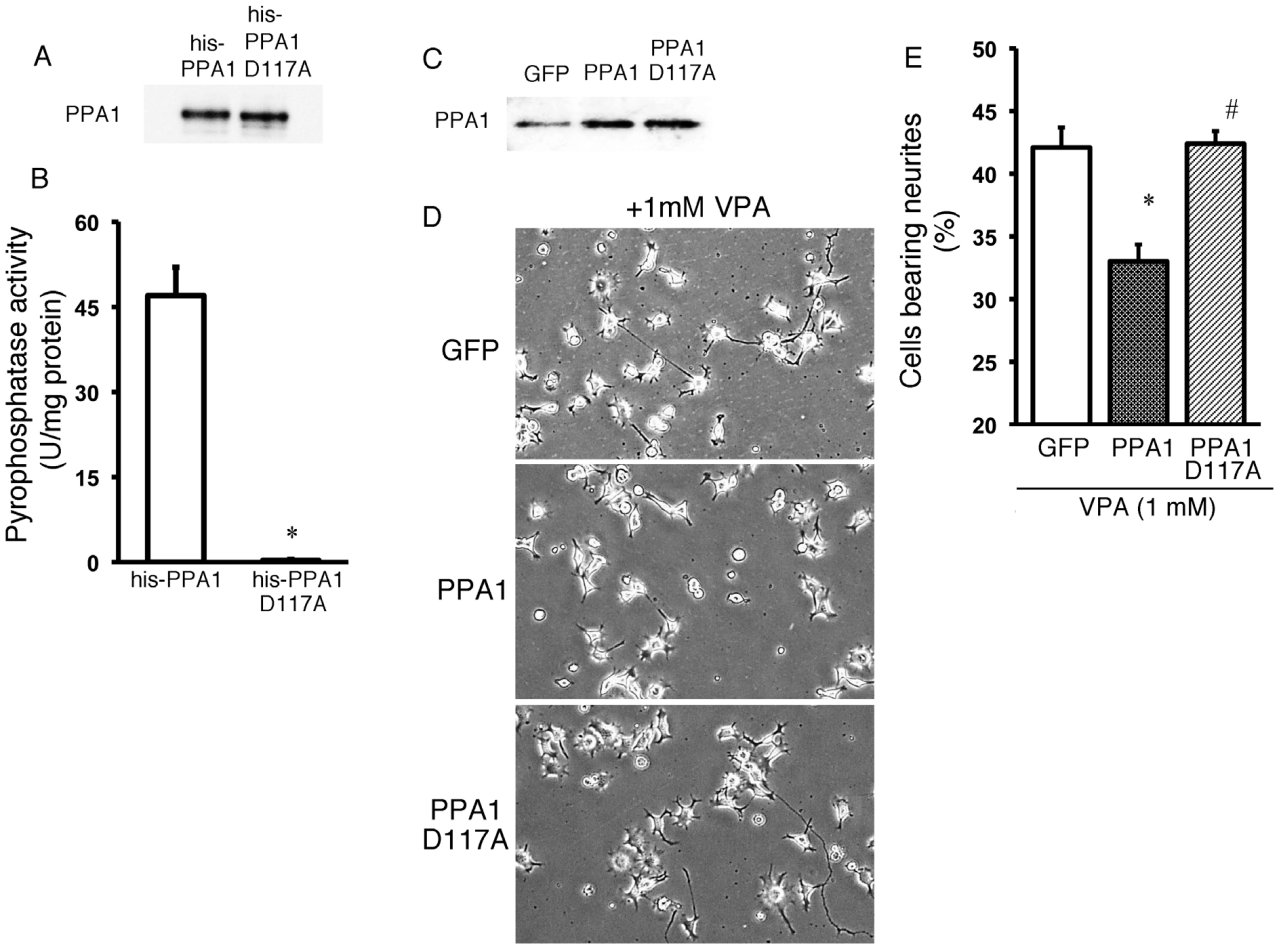
Role of pyrophosphatase activity on PPA1-induced inhibition of neuronal differentiation in N1E115 cells. (A) Recombinant wild-type PPA1 (his-PPA1) and PPA1 Asp117Ala (his-PPA1 D117A) proteins. Western blot using anti-PPA1 antibody showed that the his-PPA1 and his-PPA1 D117A protein amount is similar. (B) Recombinant protein pyrophosphatase activity. Pyrophosphatase activity was seen in his-PPA1 while no activity was detected in his-PPA1 D117A (n = 5). (C) Effect of either green fluorescence protein (AD-GFP), wild-type PPA1 (AD-PPA1), or PPA1 D117A (AD-PPA1 D117A) overexpression using adenoviral vector, on PPA1 protein levels in N1E115 cells treated with 1 mM valproic acid (VPA). (D) Typical morphological changes in N1E115 cells treated with VPA, using AD-GFP overexpression as a control (VPA+GFP), AD-PPA1 (VPA+AD-PPA1) or D117A (VPA+AD-PPA1 D117A). (E) Quantitative analysis of the neurite growth ratio is shown. Neurite growth is determined by morphological analysis as described in the Experimental procedure (n = 100). *p<0.05 vs. his-PPA1, #p<0.05 vs. N1E115 cells treated with VPA+GFP, a<0.05 vs. VPA+Ad-PPA1.

To determine the role of PPA1 in neurite growth, an amino acid mutation was made in mouse PPA1. Aspartic acid at the position 117 in PPA1 is critical for pyrophosphatase activity [21], [22], and thus, replacing the aspartic acid with an alanine resulted in an inactive pyrophosphatase form. To confirm the effect of the PPA1 mutation, recombinant wild-type PPA1 and D117A PPA1 proteins were produced. The same amount of recombinant wild-type PPA1 protein and D117A PPA1 protein was detected (Figure 3A), while pyrophosphatase activity was only detected in wild-type PPA1 (Figure 3B). These results indicate that the D117A mutation in PPA1 led to its inactivation as a pyrophosphatase (Figure 3A and B).

We then generated an adenovirus vector containing PPA1 D117A to test whether or not the pyrophosphate activity in PPA1 was required to inhibit neurite growth. The results of either PPA1 or PPA1 D117A overexpression in N1E115 cells treated with VPA, an activator of neurite growth, are shown in Figures 3C to E. An increase in the PPA1 protein level was observed following treatment with the adenoviral vector containing wild-type PPA1 and PPA1 D117A compared to that containing GFP (Figure 3C). Wild-type PPA1 overexpression can inhibit neurite growth in N1E115 cells, while no inhibitory effect was detected by overexpression of PPA1 D117A, a pyrophosphatase inactive protein (Figure 3D and E). These results indicate that PPA1 pyrophosphatase activity is necessary for neurite growth inhibition in N1E115 cells.

### Underlying PPA1 signals in N1E115 cells

**Figure 4:**
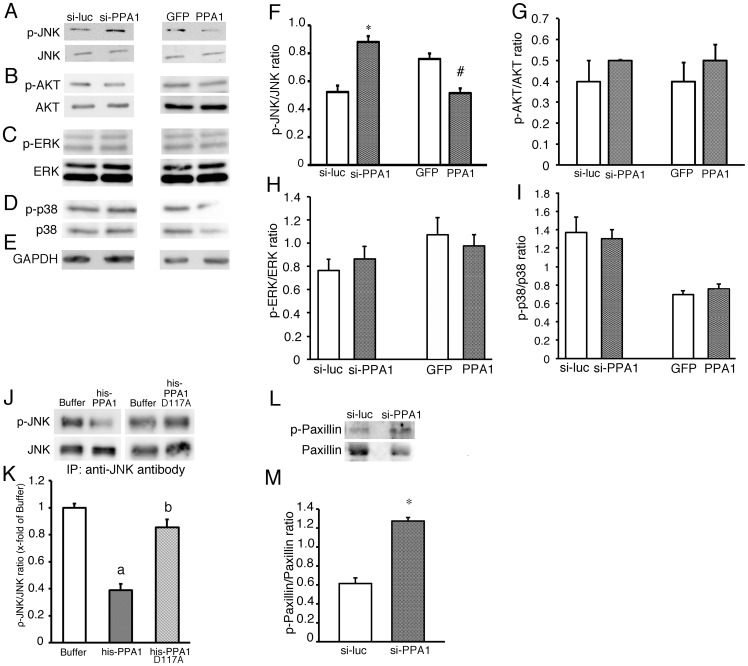
Phosphorylated protein kinase levels in N1E115 cells. (A–E) Representative Western blots using anti-phospho-JNK and JNK (A), phospho-AKT and AKT (B), phospho-ERK and ERK (C), phospho p38 and p38 (D) and GAPDH (E). N1E115 cells were treated with si-RNA targeted to mouse PPA1 (si-PPA1) or treated with adenovirus containing mouse PPA1 (PPA1). Si-RNA targeted to luciferase (si-luc) was used as a si-PPA1 control and adenovirus containing GFP (GFP) was used as a control for adenovirus containing mouse PPA1. (F–I) Quantitative analysis of the phosphorylated JNK/JNK ratio (F), pAKT/AKT ratio (G), pERK/ERK ratio (H) and p-p38/p38 ratio (I) are shown. (J and K) Direct effects of recombinant PPA1 (his-PPA1) or PPA1 D117A (his-PPA1 D117A) proteins on the phosphorylated JNK level immunoprecipitated from N1E115 cells. As a control, buffer without the recombinant protein was added (buffer). (n = 5) Representative Western blot using anti-phospho-JNK and JNK (J) and quantitative analysis of the phosphorylated JNK/JNK ratio (K) (n = 5). (L and M) Phosphorylated paxillin level in N1E115 cells. Representative Western blot using anti-phospho-paxillin and paxillin (L) and quantitative analysis of the phosphorylated paxillin/paxillin ratio (M) (n = 5). *p<0.05 vs. si-luc, ^#^p<0.05 vs. GFP, ^a^<0.05 vs. buffer, and b<0.05 vs. his-PPA1.

PPA1 is thought to play a role in catalyzing the hydrolysis of pyrophosphates into organic phosphates [15]. However, no alteration of cell proliferation was detected by knockdown and overexpression of PPA1 in N1E115 cells. Thus, we hypothesized that PPA1 may inhibit neurite growth by inactivating the signaling enzyme via dephosphorylation. To examine this hypothesis, we measured the phosphorylation level of protein kinases such as JNK, ERK, P38 MAP kinase and AKT, which are known to play an important role in neurite growth (Figure 4A to I) [11], [30], [31]. PPA1 knockdown increased the level of phospho-JNK, while PPA1 overexpression decreased it (Figure 4A) and no alteration of phospho-specific antibody signals of other kinases such as ERK and AKT were seen in N1E115 cells (Figure 4B to E, and G to I). These results suggest that PPA1 can modulate the phosphorylation status of JNK in N1E115 cells. Although PPA1 can dephosphorylate the JNK, it is still uncertain whether it is a direct or an indirect effect via PPA1. To address this, an immunoprecipitation assay using anti-JNK antibody was performed (Figure 4J and K). Phospho-JNK was detected in the immunoprecipitated N1E115 lysate using anti-JNK antibody (Figure 4J and K). The JNK phosphorylation level was suppressed by addition of recombinant wild-type PPA1, while no effect was seen using recombinant PPA1 D117A, the inactive form of pyrophosphatase (Figure 4J and K). These results suggest that PPA1 can dephosphorylate the phosphorylated-JNK.

Since a previous study showed that phosphorylation of paxillin by JNK is critical for neurite growth in N1E115 cells [11], the paxillin phosphorylation level was measured in N1E115 cell treated with si-PPA1 (Figure 4L and M). The phospho-paxillin level was elevated by PPA1 knockdown (Figure 4L and M). Thus, PPA1 can dephosphorylate JNK in the JNK/paxillin cascade and inactivate it concomitant with enhancing neurite growth in N1E115 cells.

### Role of PPA1 in primary rat cortical neurons

**Figure 5:**
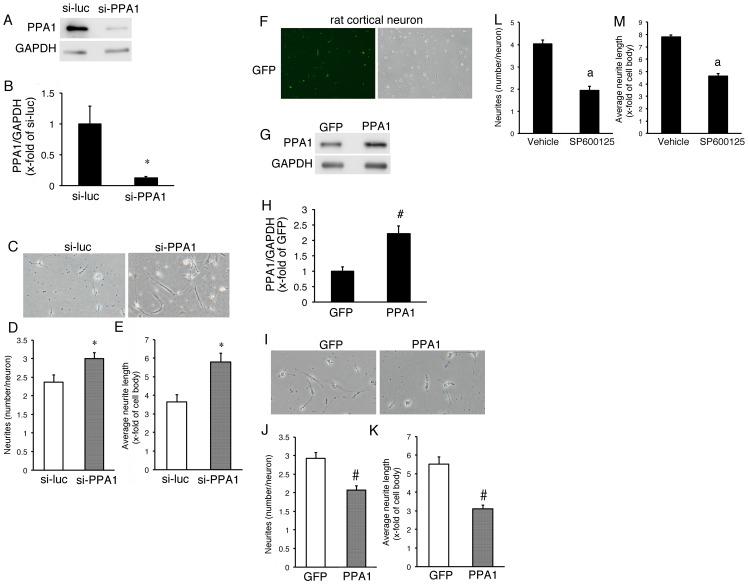
Modification of PPA1 in rat cortical neurons. (A) Representative Western blot analysis (A) and quantitative analysis of PPA1 protein (B). PPA1 knock-down in rat cortical neurons using siRNA targeted to rat PPA1 (si-PPA1). As a control, si-RNA targeted to luciferase was used (si-luc) (n = 5). (C) A representative rat cortical neuron treated with si-RNA targeted to rat PPA1 and targeted to luciferase as a control. (D–E) Quantitative analysis of neurite growth in rat cortical neurons. Neurite growth is determined by morphological analysis as described in the Experimental procedure (n = 100). (F) GFP overexpression in rat cortical neurons using adenovirus containing GFP. (G and H) Representative Western blot analysis (G) and quantitative analysis of PPA1 protein (H). PPA1 overexpression in rat cortical neurons using adenovirus containing mouse PPA1 (PPA1). As a control, adenovirus containing GFP was used (GFP) (n = 5). (I) Representative rat cortical neurons treated with PPA1 adenovirus and GFP virus as a control. (J and K) Quantitative analysis of neurite growth from rat cortical neurons (n = 100). (L and M) A representative rat cortical neuron treated with 10 µM SP600126, an inhibitor of JNK (SP600125), and vehicle as a control (vehicle) (n = 5).*p<0.05 vs. si-luc, ^#^p<0.05 vs. GFP. ^a^p<0.05 vs. vehicle.

PPA1 can act as a JNK protein phosphatase, concomitant with enhancement of the neurite growth in N1E115 cells. Next, we examined the role of PPA1 in primary neurons. A rat cortical neuron was isolated and cultured. In the present experimental condition, neurite growth usually occurred in the most of the cells. Thus, we measured the number of neurites growing from the neuron and the average neurite length (Figure 5D and E, J and K, L and M). PPA1 protein levels were dramatically reduced by treatment with the PPA1-specific siRNA compared to that in the neuron treated with the luciferase-specific siRNA (Figure 5A and B). All parameters associated with neurite growth and neuronal differentiation were enhanced by knockdown of PPA1 in the rat cortical neuron (Figure 5B–E). In our pilot study using an adenoviral vector containing GFP, approximately 70% of rat cortical neurons were infected and expressed the GFP gene 12 hr after infection (Figure 5F). All parameters associated with neurite growth and neuronal differentiation were attenuated by overexpression of PPA1 in the rat cortical neuron (Figure 5I–K). Neurite growth was inhibited by treatment with the JNK inhibitor, SP600125 (Figure 5L and M). These data suggest that, similar to the N1E115 neuroblastoma cell line, PPA1 can inhibit neuronal differentiation such as neurite growth in the rat primary neuron, possibly via JNK dephosphorylation.

## Discussion

ATP hydrolysis releases pyrophosphate, which becomes a metabolic inhibitor at high concentrations in cells and must be hydrolyzed immediately to facilitate the biosynthesis of various macromolecules [32]. It is known that PPA1 is an enzyme (EC 3.6.1.1) that catalyzes the conversion of one molecule of pyrophosphate to two phosphate ions [15], [16]. Therefore, PPA1 may play a role in the thermodynamic driving force for several important biosynthetic reactions in yeast [17], bacteria [18] and plants [32]. Although these results suggest that PPA1 can act as a pyrophosphatase in some cell types, the functional role of PPA1 as a pyrophosphatase or its other functions in neuronal cells remains uncertain. In the present study, we examined the functional role of PPA1 in the neuronal cell using the neuroblastoma cell line, N1E115. No alteration of cell proliferation was detected using PPA1 modification in N1E115 cells. This result may suggest that changes in DNA or RNA synthesis by the PPA1 modification are unlikely in N1E115 cells. In contrast, PPA1, as a protein phosphatase, can inhibit neurite growth in N1E115 cells by alteration in the JNK phosphorylation level, and this effect was also observed in the rat dorsal root ganglion [12]. Thus, while the contribution of PPA1-induced pyrophosphate degradation on neurite growth cannot be ruled out, PPA1 may play an important role in neurite growth, possibly by direct inactivation through JNK dephosphorylation.

JNK was initially identified as the kinase phosphorylating the transcription factor c-Jun and related members; there is now evidence demonstrating that neurogenesis and neuritogenesis require phosphorylation of c-Jun and these related members [33]. JNK also has several important substrates among the actin and tubulin cytoskeletal proteins [33]. Among these JNK substrates, the focal adhesion adaptor protein, paxillin, is a particularly promising JNK substrate candidate [11], [12]. It is known that paxillin, a multifunctional focal adhesion protein, is phosphorylated at ser 178 by JNK, and that this phosphorylation is critical for neurite extension to occur [11]. Thus, JNK dephosphorylation by PPA1 can result in a decrease in phosphorylated paxillin and the alteration of paxillin may play an important role in neurite growth in N1E115 cell and rat cortical neurons.

PPA1 knockdown caused an increase in JNK phosphorylation without an increase in ERK phosphorylation, and JNK signal is critical for neurite growth in N1E115 cells [11], [12]. In addition, PPA1 overexpression can attenuate neurite growth stimulated by VPA treatment. A previous study showed the effects of VPA on the activation of the ERK pathway in E18 cortical neurons [34]. Other studies also showed that VPA promoted neurite growth and cell reemergence in an ERK pathway-dependent manner [35]–[37]. However, another study showed that ERK inhibition with U0126, a specific ERK inhibitor, was not able to inhibit VPA-induced neuronal differentiation [38]. We also showed that stimulation of the JNK cascade plays a critical role in N1E115 cell neurite extensions following treatment with VPA [12]. Thus, multiple signaling pathways are involved in VPA-induced neurite growth and neuronal differentiation of neural progenitor cells.

## Conclusion

We examined the role of PPA1 in neuronal differentiation using the loss and gain of function analysis in the mouse neuroblastoma cell line, N1E115. PPA1 inhibited neurite like growth in N1E115 cells without changing cell proliferation. PPA1 can enhance the JNK dephosphorylation level without changing the phosphorylation of other signaling kinases such as ERK and AKT. PPA1 can inhibit neuronal differentiation such as neurite growth in the rat primary neuron. PPA1 may play a role in neuronal differentiation via JNK dephosphorylation, but the contribution of pyrophosphate degradation by PPA1 in neurite growth cannot be ruled out.
